# AAV-Mediated Clarin-1 Expression in the Mouse Retina: Implications for USH3A Gene Therapy

**DOI:** 10.1371/journal.pone.0148874

**Published:** 2016-02-16

**Authors:** Astra Dinculescu, Rachel M. Stupay, Wen-Tao Deng, Frank M. Dyka, Seok-Hong Min, Sanford L. Boye, Vince A. Chiodo, Carolina E. Abrahan, Ping Zhu, Qiuhong Li, Enrica Strettoi, Elena Novelli, Kerstin Nagel-Wolfrum, Uwe Wolfrum, W. Clay Smith, William W. Hauswirth

**Affiliations:** 1 Ophthalmology, University of Florida, Gainesville, FL, United States of America; 2 Cell and Matrix Biology, Institute of Zoology, Johannes Gutenberg-University of Mainz, Mainz, Germany; 3 CNR Neuroscience Institute, Pisa, Italy; Dalhousie University, CANADA

## Abstract

Usher syndrome type III (USH3A) is an autosomal recessive disorder caused by mutations in clarin-1 *(CLRN1)* gene, leading to progressive retinal degeneration and sensorineural deafness. Efforts to develop therapies for preventing photoreceptor cell loss are hampered by the lack of a retinal phenotype in the existing USH3 mouse models and by conflicting reports regarding the endogenous retinal localization of clarin-1, a transmembrane protein of unknown function. In this study, we used an AAV-based approach to express CLRN1 in the mouse retina in order to determine the pattern of its subcellular localization in different cell types. We found that all major classes of retinal cells express AAV-delivered CLRN1 driven by the ubiquitous, constitutive small chicken β-actin promoter, which has important implications for the design of future USH3 gene therapy studies. Within photoreceptor cells, AAV-expressed *CLRN1* is mainly localized at the inner segment region and outer plexiform layer, similar to the endogenous expression of other usher proteins. Subretinal delivery using a full strength viral titer led to significant loss of retinal function as evidenced by ERG analysis, suggesting that there is a critical limit for CLRN1 expression in photoreceptor cells. Taken together, these results suggest that CLRN1 expression is potentially supported by a variety of retinal cells, and the right combination of AAV vector dose, promoter, and delivery method needs to be selected to develop safe therapies for USH3 disorder.

## Introduction

Usher syndrome (USH) is an autosomal recessively inherited disorder responsible for more than half of combined deafness and blindness in humans [1, 2]. It represents a group of clinically and genetically heterogeneous disorders, classified into three major types, USH1, USH2, and USH3, depending on the onset and severity of the symptoms, and the presence or absence of vestibular function [3–6]. It affects both photoreceptors and cochlear hair cells, the sensory neurons of the retina and inner ear, respectively. Usher syndrome type III (USH3) is caused by clarin-1 **(***CLRN1*) gene mutations [7–9], leading to progressive sensorineural hearing loss and retinal degeneration, with variable vestibular dysfunction [10–12]. Vision loss is due to a relatively slow-onset rod dysfunction, with progressive degeneration of photoreceptor cells [13]. Peripheral rod function is lost first, followed by a relatively slow decline of central cone function that extends for decades, resulting in progressively constricted visual fields and impaired visual acuity, consistent with the presentation of retinitis pigmentosa [14, 15]. Although USH3 is relatively rare compared to USH1 and 2, it is responsible for a large proportion of cases in the Finnish and Ashkenazi Jewish populations, where it accounts for approximately 40% of USH patients [13, 16]. Cochlear implantation addresses to some degree the hearing impairment, but there is no treatment that prevents the progressive photoreceptor degeneration leading to vision loss.

The proteins encoded by USH genes are thought to form an interactive network based on co-localization and protein binding studies [17, 18]. They belong to different network classes with diverse functions [19], such as the actin-based motor protein myosin VIIa (USH1B), the cell adhesion proteins, cadherin 23 (USH1D) and protocadherin 15 (USH1F), the transmembrane usherin (USH2A), the G-protein coupled receptor VLGR1 (USH2C), and the scaffold proteins harmonin (USH1C), SANS (USH1G), and whirlin (USH2D). The major sites for co-localization of USH proteins are the connecting cilium and inner segments in photoreceptors, and the stereocilia of hair cells in the cochlea [20, 21]. In addition, some of these proteins are also found at ribbon synapses of both sensory cells [18, 22–24]. Mutations affecting a particular USH member are thought to destabilize other protein interactions within the network [25]. Many USH proteins provide direct or indirect links to F-actin filaments and microtubules, two key components of the cytoskeleton [17, 26, 27]. The existence of an “usher interactome”, a dynamic protein complex present within both photoreceptors and cochlear hair cells, provides a potential explanation for the shared phenotype produced by mutations in a variety of USH genes encoding proteins with different functions [20].

CLRN1 is predicted to have four transmembrane domains similar to the tetraspanin family, with intracellular amino- and carboxy-terminal tails, based on its primary structure [9]. Tetraspanins are involved in a wide range of molecular interactions affecting cell signaling, adhesion, morphology and protein trafficking [28]. They act as membrane organizers by interacting with other membrane proteins, forming tetraspanin-enriched microdomains, and recruiting binding partners such as signaling receptors and adhesion proteins. A potential role for CLRN1 in the regulation of actin filaments was uncovered by Tian et al. using cell-culture assays, strengthening the hypothesis that this protein may belong to the USH protein network [29].

The most common USH3 mutation in North America is CLRN1 N48K [16]. Cell culture experiments demonstrated that N48K mutant CLRN1 lacks the position 48 glycosylation site, fails to localize to the plasma membrane, and accumulates mainly in the endoplasmic reticulum [29, 30]. Expression of CLRN1 in transfected mouse cochlear cells *in vitro* determined that the protein is essential for the morphogenesis and maintenance of hair bundle stereocilia, and that mutant N48K fails to localize to the bundle [31]. Moreover, the F-actin-rich hair cell stereocilia in *Clrn1* knock-out (KO) mice are poorly developed and disorganized [32, 33]. Both *Clrn1* KO and the N48K knock-in mice display an early onset hearing loss, but normal retinal morphology and function [33]. It has been suggested that the lack of a retinal phenotype in these and other USH mouse models is due to the existence of redundant mechanisms compensating for the lack of functional USH protein, to the short life span of the mouse, and/or to morphological differences between human and mouse photoreceptors, perhaps because mice lack well-developed, actin-filled calycal processes at the apical region of inner segments [33, 34].

Previous studies of CLRN1 localization concluded that CLRN1 is difficult to detect either because its endogenous expression levels are low or its potential antibody binding epitopes are blocked by other binding partners, preventing a reliable identification of its cellular localization in the retina [33]. One immunohistochemistry study showed that the protein is present in mouse photoreceptor cells [35], while other researchers concluded that its mRNA originates exclusively from the inner retina [33]. In this work, we used an adeno-associated vector (AAV) to examine *in vivo* the preferred subcellular localization of CLRN1 in a variety of cell types following intravitreal or subretinal delivery in adult mice. Although this approach does not identify the specific retinal cell type(s) where CLRN1 is endogenously produced, the subcellular localization of AAV-expressed CLRN1 should provide useful information about its function. In addition, since light-driven translocation of phototransduction proteins was previously used to detect retinal defects in several mouse models of retinal disease [36–39], we also investigated whether *Clrn1* KO mice displayed any abnormalities in the compartmentalization of arrestin-1 and α-transducin following light exposure.

## Materials and Methods

### Ethics Statement and Animals

The generation and characterization of *Clrn1* KO mice have been previously described [33]. Adult *Clrn1* KO and corresponding age-matched wild-type isogenic controls (at least 3-months of age), as well as adult C57BL/6 mice were used in all experiments. Animals were maintained on a cycle of 12 hour of light (200 lux) and 12 hour of darkness in the University of Florida Health Science Center Animal Care Services Facility (Gainesville, FL). All experiments were approved by University of Florida Institutional Animal Care and Use Committees and conducted in accordance with the Association for Research in Vision and Ophthalmology (ARVO) Statement for the Use of Animals in Ophthalmic and Vision Research.

### Preparation and Delivery of the CLRN1-AAV vector

A self-complementary AAV2 quadruple capsid tyrosine mutant (Y272,444,500,730F) vector (scAAV2quadYF) was used for packaging either a C-terminal Venus-tagged human clarin-1 (*CLRN1*-Venus, from Dr. Imanishi at Case Western University) or an HA-tagged *CLRN1* cDNA under the control of the ubiquitous smCBA (small chicken β-actin) promoter. An AAV vector was also generated containing the reporter mCherry driven by a proximal 2000 nucleotide mouse *Clrn1* promoter region [40]. The viral vectors were produced and purified according to previously reported methods [41]. Viral titer was determined by real-time PCR. Subretinal and intravitreal injections were performed in adult wild-type mice under anesthesia as described [42, 43]. In brief, the nasal cornea was penetrated with a 30.5-gauge disposable needle and a 33-gauge unbeveled, blunt-tip needle on a Hamilton syringe was introduced into the subretinal space. For intravitreal injections, a 33-gauge beveled needle was passed through the sclera, at the equator, next to the limbus, and into the vitreous. Each right eye received 1 μl of AAV vector (approximately 1x10 ^13^ genome copies/ml), while the left eye remained uninjected. At approximately 6 to 8 weeks post-injection, the CLRN1-Venus was detected directly by visualization of Venus (a variant of YFP) fluorescence in retinal sections, while the CLRN1-HA tagged protein was visualized by immunostaining with a high-affinity anti-HA-Fluorescein conjugated antibody (3F10, Roche Diagnostics, Indianapolis, IN). A mouse monoclonal antibody (B6-30) against rod opsin [44] was used to label the outer segments.

### Electroretinographic analysis (ERG)

Treated C57BL/6 mice were dark-adapted (>12 hr) and anesthetized with a mixture of ketamine (72 mg/kg)/xylazine (4 mg/kg) by intraperitoneal injection, and ERG experiments were conducted at 2 months following subretinal injections with the AAV2 quad YF vector carrying the *CLRN1*-HA-tagged transgene as described above. For toxicity assays, one eye in each mouse received one microliter of either a full strength titer of approximately 10^13^ vector genome/ml (vg/mL) or serially diluted vectors of 10^12^, 10^11^, or 10^10^ vg/mL. Pupils were dilated with topical agents (1% atropine sulfate, 2.5% phenylephrine hydrochloride) under dim red light. Full-field ERGs were recorded as previously described [42], using a UTAS Visual Diagnostic System (LKC Technologies, Gaithersburg, MD). Dark-adapted ERGs were elicited with 0.02, 0.2 and 2 scot-cd·sec·m^–2^ stimuli. Differences in maximum b-wave amplitudes between AAV *CLRN1*-HA -injected and uninjected contralateral left eyes were analyzed by Student *t* test (GraphPad Prism 6.0, GraphPad Software, San Diego, CA), and considered statistically significant if p<0.05. All ERG data are presented as mean ± SEM.

### Light-driven translocation experiments and immunohistochemical analysis

All mice were dark adapted for at least 12 hours prior to the light adaptation. Dim red light was used for all procedures in the dark room. For light exposure, unanesthetized *Clrn1* KO and wild-type mice were placed in a clear plastic box and exposed to light (1000 lux intensity at the cage level) for 1 hour. Following euthanasia, the eyes were enucleated, placed into vials containing 4% paraformaldehyde solution, and fixed for at least 4 hours prior to moving them into 1× phosphate-buffered saline (PBS). The eyes were processed for paraffin embedding and sectioned at a thickness of 4 μm, at the same retinal locations for both wild-type and knock-out eyes. For immunostaining, deparaffinized tissue sections were dewaxed in xylene and rehydrated in a graded series of ethanol, and then incubated with 0.5% Triton X-100 for 15 min, followed by blocking with a solution of 1% albumin for 1 hour. Wild-type and *Clrn1* KO retinal sections were processed simultaneously under identical conditions. Arrestin-1 was detected either with mouse monoclonal antibody SCT-128 [45], or C10C10 [46] at 1:200 dilution. The α-transducin subunit was detected using a rabbit polyclonal antibody (sc-389, Santa Cruz Biotechnology, Dallas, TX) at 1:1000 dilution. Secondary antibodies conjugated to Alexa-594 or Alexa-488 fluorophore (Molecular Probes/Invitrogen, Eugene, OR) were used at 1:500 dilution in PBS. All sections were examined by fluorescence microscopy, using either a Leica TCS SP2 laser scanning confocal microscope (Leica, Heidelberg, Germany) or an Axiophot microscope (Zeiss, Thornwood, NY). Vertical densitometric profile plots representing the average fluorescent signal intensity across the photoreceptor layer were generated with ImageJ's Plot Profile function for both arrestin-1 and α-transducin subunit, using the DAPI nuclear stain to determine the outer plexiform layer (OPL) edge as described [47].

### Statistical Analysis

The fluorescence intensity of the immunolabeled proteins was analyzed as previously described [48, 49]. ImageJ software (National Institutes of Health) was used for measuring the relative intensity of the fluorescence signal in at least three different randomly located regions from the mid-central retinas of wild-type and *Clrn1* KO mice. The relative signal intensity in the OPL was expressed as a percentage of the total intensity in the photoreceptor layer. Statistical differences between the two groups were assessed by unpaired *t* test (GraphPad Prism 6.0, GraphPad Software, San Diego, CA). Error bars represent the mean ± standard error of mean (SEM). Results are considered statistically significant if *p<0.05.

### Electron microscopy

For ultrastructural analysis of murine photoreceptor cells, enucleated eyes from wild-type and *Clrn1* KO mice were fixed in 2.5% glutaraldehyde in 0.1 M cacodylate buffer containing 0.1 M sucrose for 1.5 h at 4°C in total. After 30 min fixation the cornea and lens were removed and the eye cups were fixed for additional 1 h. Subsequently, eye cups were washed with 0.1 M cacodylate buffer containing 0.1 M sucrose for 30 min. Next, eye cups were fixed with 2% osmium tetroxide in 0.1 M cacodylate buffer containing 0.1 M sucrose for 1 h at room temperature, followed by dehydration in ethanol (30–100%) and embedding in Renlam M-1 resin (Serva Electrophoresis, Heidelberg, Germany). Ultrathin sections, counterstained with ethanolic uranyl acetate and lead citrate, were analyzed in transmission electron microscope (Tecnai 12 BioTwin, FEI, Netherlands). Images were obtained with a CCD camera (charge-coupled-device camera; SIS MegaView3; Surface Imaging Systems, Herzogenrath, Germany) and processed with Adobe Photoshop CS (Adobe Systems).

## Results

**Fig 1:**
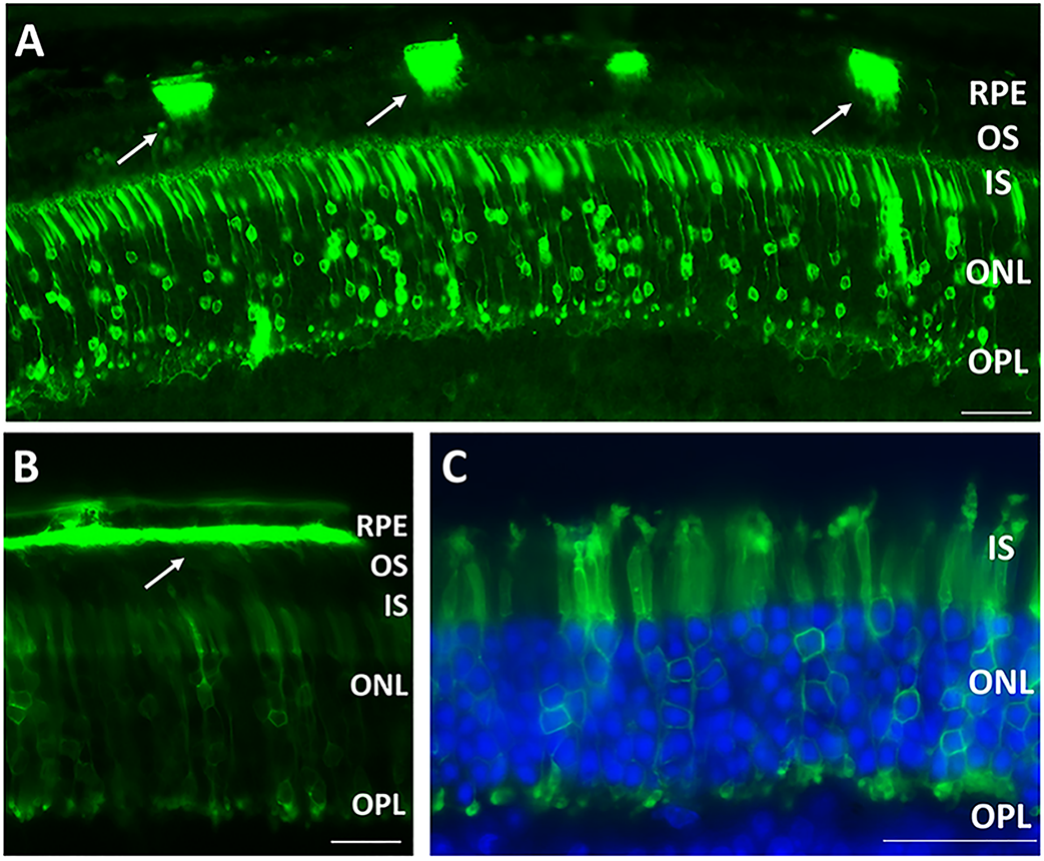
Localization of vector-expressed CLRN1-Venus following subretinal delivery. A. CLRN1-Venus fusion fluorescence was detected on the apical side of RPE cell membranes (arrows), and in photoreceptor IS, ONL and OPL. Scale bar: 20 μm. Panel B shows a continuous area of intense apical RPE CLRN1-Venus (arrow). C. Higher magnification image showing strong CLRN1-Venus fluorescence in specific regions within photoreceptor cells: IS, cell body membrane and OPL. Nuclei are stained blue with DAPI. Scale bar: 30 μm. Abbreviations: RPE, Retinal Pigment Epithelium; IS, inner segment; OS, outer segment; ONL, outer nuclear layer; OPL, outer plexiform layer; INL, inner nuclear layer.

**Fig 2:**
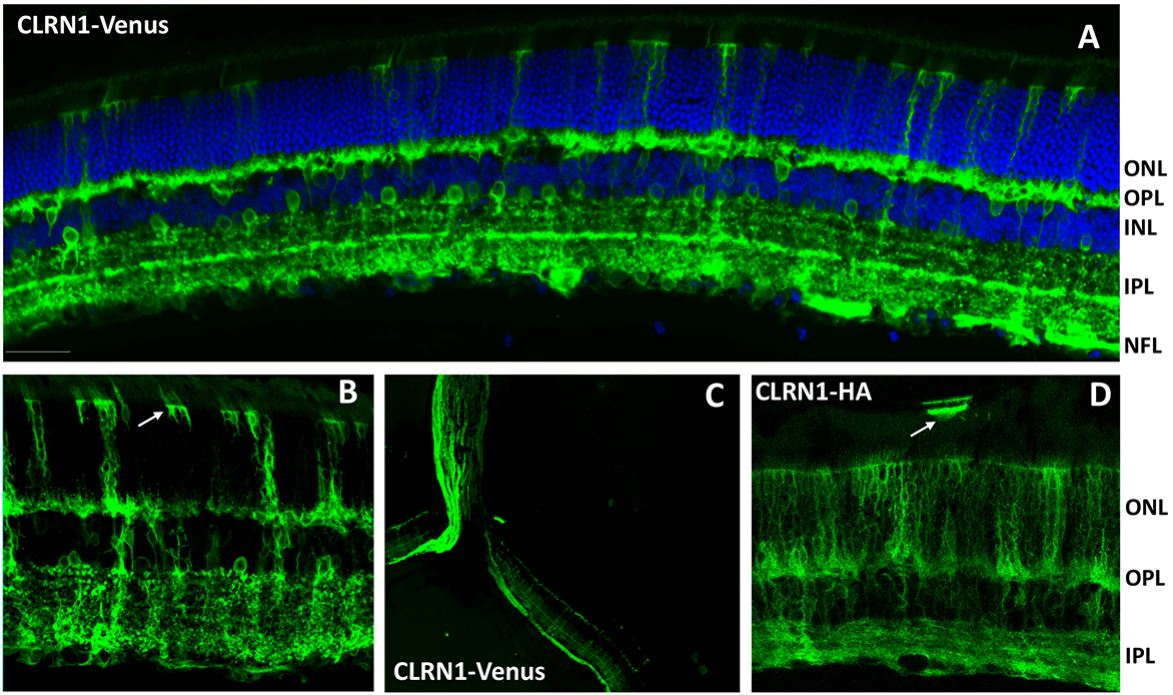
Localization of vector-expressed CLRN1 following intravitreal delivery. A. Representative image of retinal cross-section showing CLRN1-Venus fusion fluorescence. Strong expression is seen at the OPL, the stratified dendrites within the IPL, and inner retinal neurons. Scale bar: 30 μm. B. Higher magnification view (40X) of CLRN1-Venus expression showing intense fluorescence at the Müller cells and their apical processes at the outer limiting membrane (arrow). C. Low magnification (10X) image showing the localization of CLRN1-Venus expression along the nerve fiber layer and optic nerve extension of ganglion cell axons. D. Localization of vector-expressed CLRN1-HA protein by immunostaining with an anti-HA antibody (40X magnification). Note the expression in numerous Müller cells with fine processes extending between photoreceptors inner segments. An isolated patch of RPE cells with strong labeling of the apical processes is also present (arrow). Abbreviations: ONL, outer nuclear layer; OPL, outer plexiform layer; IPL, inner plexiform layer; NFL, nerve fiber layer.

An AAV2 quad YF vector carrying a *CLRN1*-Venus transgene cDNA driven by a smCBA promoter (scAAV2quadYF-smCBA-*CLRN1*-Venus) was delivered to adult wild-type mouse eyes via either the subretinal or intravitreal route in order to examine the pattern of CLRN1 distribution by direct fluorescence imaging. We determined that CLRN1 expression is supported by a variety of cells, including RPE, rod and cone photoreceptors, amacrine, bipolar, horizontal, ganglion and Müller cells (Figs 1 and 2). Of these, photoreceptors have a unique compartmentalized morphology that makes them amenable for sub-cellular localization studies. Subretinal delivery, which primarily targets photoreceptors and the RPE, led to robust CLRN1-Venus protein expression at the inner segments, outer nuclear cell body membrane and the outer plexiform layer (Fig 1).

**Fig 3:**
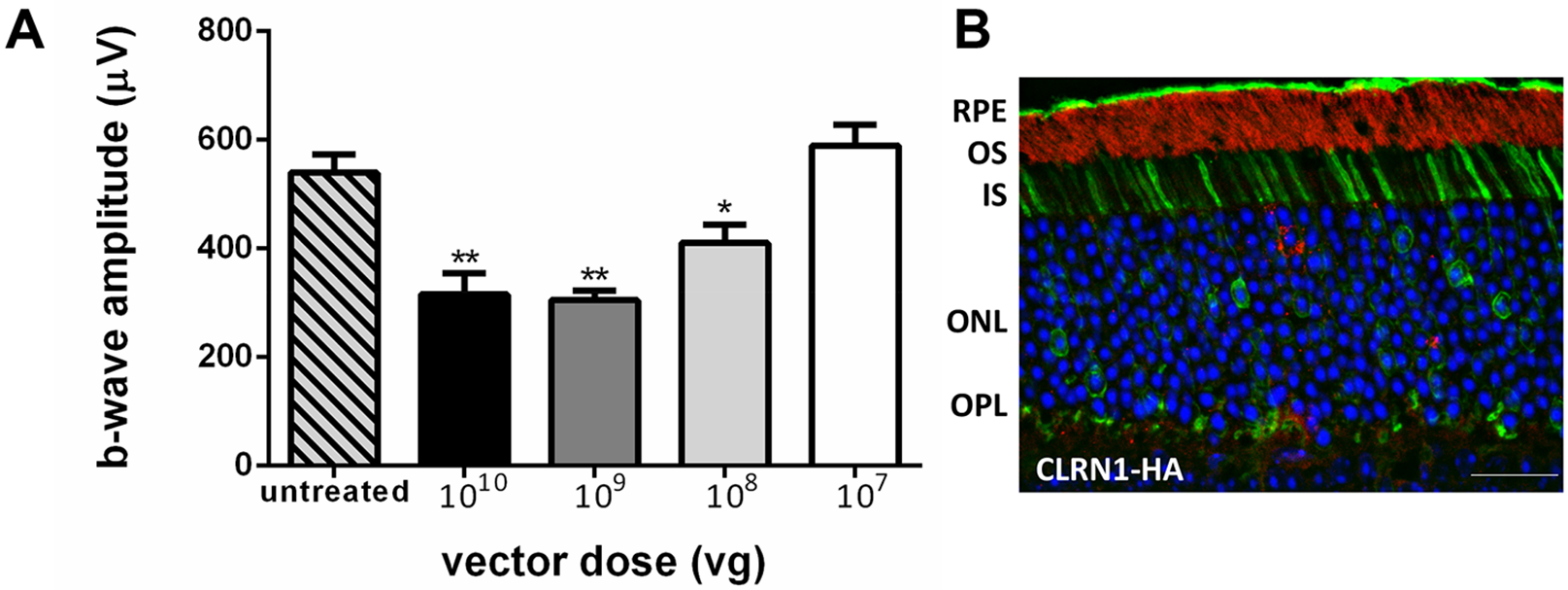
Evaluation of retinal function and CLRN1-HA expression following subretinal delivery. A. Bar graphs show the average maximum ERG b-wave amplitudes in scotopic, dark-adapted conditions of untreated wild-type control eyes, compared to AAV-treated eyes that received decreasing doses of AAV-CLRN1-HA vector (2 months post-injection). Maximum b-wave amplitudes in AAV-treated eyes were dose dependent, and were significantly lower than untreated controls at 10^10^, 10^9^, and 10^8^ vg (*, p<0.05, **, p<0.001). B. Localization of CLRN1-HA protein following subretinal delivery of the AAV2quad smCBA vector. CLRN1-HA fluorescence (green) was detected by immunohistochemistry in photoreceptor IS region, ONL and OPL, and was absent from the outer segments (labelled with a rhodopsin antibody, red). The eyes received diluted vector (10^8^ vg). Scale bar: 20 μm.

Strong CLRN1-Venus fluorescence following intravitreal delivery was found in the outer and inner plexiform layers where the synaptic contacts are formed in the retina (Fig 2). CLRN1-Venus was detected in various retinal neurons, including horizontal, bipolar and amacrine cells, identified based on their location and morphology (Fig 2A). In addition, CLRN1 expression in Müller glia was apparent throughout the retina, concentrating particularly at their apical microvilli that extend upward from the outer limiting membrane, as well as in Müller cell processes surrounding the outer plexiform layer (Fig 2B). CLRN1 was also detected prominently at the nerve fiber layer formed by the ganglion cell axons and extended towards and into the optic nerve (Fig 2C). Although previous studies in cochlear hair cells showed that CLRN1 cellular localization is not affected by the presence of relatively large reporter proteins such as YFP (27kDa) on the C-terminal end of CLRN1 [31, 50], we also performed experiments in which CLRN1 was tagged with the much smaller hemagglutinin (HA) sequence and its expression was detected by immunostaining. Subretinal and intravitrealy delivered CLRN1-HA vector led to a similar pattern of expression as CLRN1-Venus. Intravitrealy delivered CLRN1-HA led to strong labeling of numerous Müller cells spanning the entire thickness of the retina, and inner retinal neurons with their extensive dendrites in the inner plexiform layer (Fig 2D). Subretinal CLRN1-HA vector led to expression at the photoreceptors inner segments, outer nuclear cell body membrane and synaptic regions, as well as the RPE apical membrane (Fig 3B).

Full field electroretinographic (ERG) analysis was performed to determine if the AAV-mediated CLRN1-HA overexpression as driven by the strong CBA promoter had potential detrimental effects on retinal function (Fig 3A). There was a significant decrease in the ERG amplitudes in treated versus untreated mouse eyes following subretinal injections with the full titer AAV2 quad YF vector carrying the *CLRN1*-HA-tagged transgene. For example, AAV-injected eyes which received a full vector dose of approximately 10^10^ vg, had a b-wave amplitude average (at 2 scot-cd·sec·m^–2^) of 315.5±39.16 μV, compared to untreated controls, with a maximum b-wave amplitude average of 539.5± 33.28μV (average ± SEM, *n* = 7, p = 0.0005). At a much lower vector dose of approximately 10^7^ vg, there was no statistically significant difference between control uninjected and AAV-CLRN1 injected eyes. Examination of subretinally injected CLRN1-HA-treated retinas following treatment with one of the lower vector doses (10^8^ vg) showed strong CLRN1 expression at the inner segment region, with weak labeling of the OPL, and normal outer nuclear layer thickness (Fig 3B).

We also tested a construct containing the 2kb region from the endogenous mouse *Clrn1* promoter driving the mCherry reporter protein following subretinal delivery. This 2000 nucleotide long CLRN1 promoter sequence is located upstream of exon 0, the translation start site of the primary CLRN1 splice variant [40]. We determined that this promoter does not restrict transgene expression specifically to either photoreceptors or RPE cells, as mCherry fluorescence was detected in both cell types (S1 Fig). However, transgene expression was low and localized mainly near the injection site, where vector particles would have had the highest concentration (S1 Fig, right panel), suggesting that this promoter is relatively weak.

**Fig 4:**
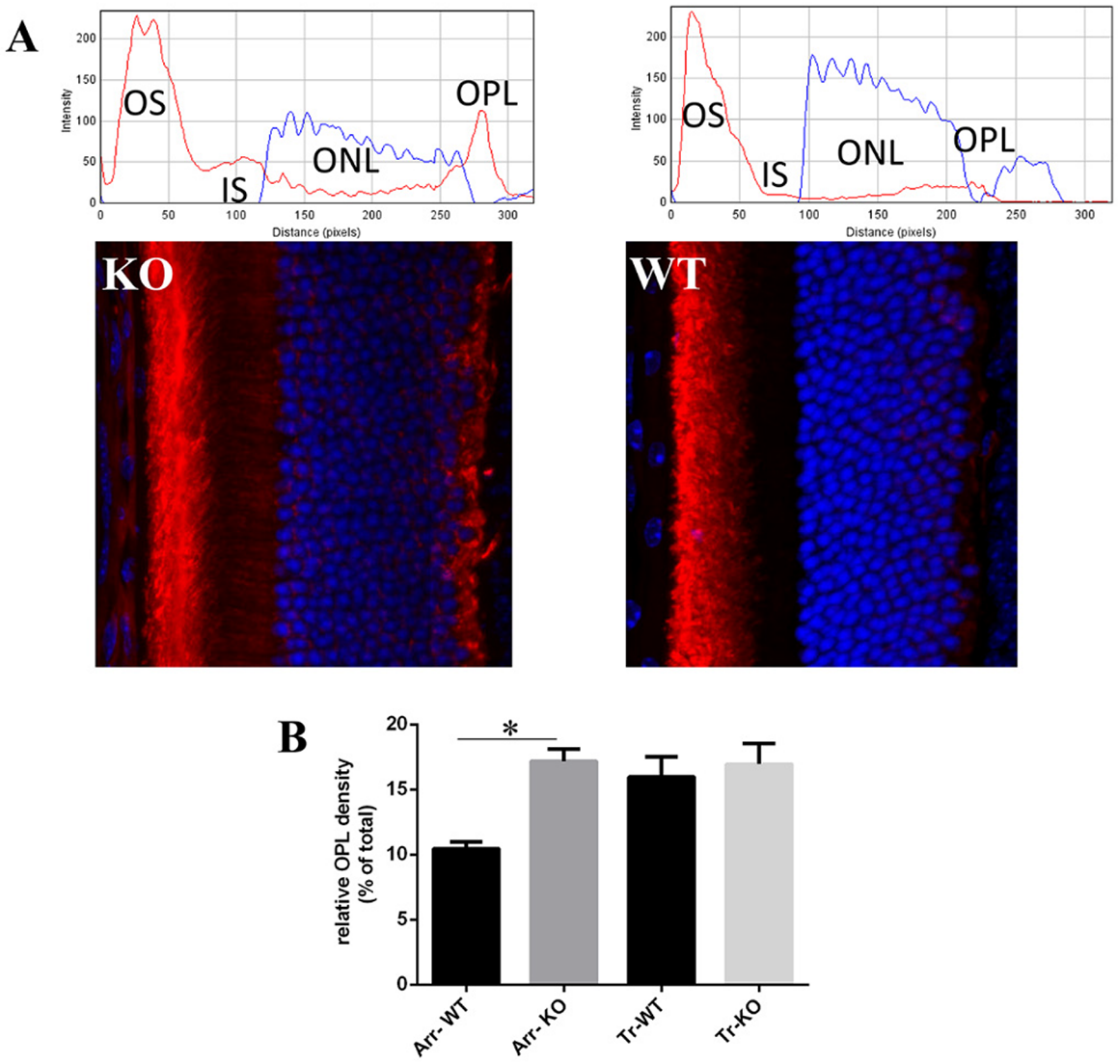
Immunofluorescent and quantification of arrestin-1 (red) in light-adapted *Clrn1* KO and WT retinal sections. A. Bottom images: Immunofluorescence distribution of arrestin-1 after light exposure translocation. Top plots: corresponding profiles of fluorescent signal intensity scanned across the photoreceptor layer. Note the increased arrestin-1 signal in the OPL of *Clrn1* KO mice (left panel) compared to WT OPL (right panel). Nuclei are stained blue with DAPI. B. Bar graph showing the relative signal intensities of arrestin-1 and α-transducin in the OPL of *Clrn1* KO and WT mice, expressed as a percentage of the total signal intensity in the photoreceptor layer (*p<0.05, n = 22 for arrestin, and n = 11 for transducin).

Since mutations in other Usher proteins have been shown to cause defects in protein translocation in response to light [37, 38], we assessed whether the lack of CLRN1 protein in KO mice leads to any abnormalities in the translocation pattern of arrestin-1 and α-transducin, which move in opposite directions between the inner and outer segments within photoreceptor cells in response to light. This movement of key phototransduction proteins through the connecting cilium plays an essential role in photoreceptor maintenance and survival, and can be used as a sensitive tool to assess subtle pathological changes in diseased photoreceptors that appear normal before degeneration starts [51, 52]. Arrestin-1 is an abundant soluble cytoplasmic protein that undergoes significant movement from the inner compartments of the photoreceptor cells towards outer segments in response to light in order to associate with light-activated phosphorylated rhodopsin with high affinity and specificity, preventing any further interaction with the G-protein transducin [53, 54]. In the dark, arrestin-1 was localized primarily to the inner segments, outer nuclear layer and synapses of photoreceptor cells, while α-transducin was found in the outer segments in both *Clrn1* KO and wild-type mouse photoreceptors (S2 Fig). In contrast, light-dependent translocation of Arrestin-1 but not α-transducin in *Clrn1* KO and isogenic wild-type controls, assessed by using previously described methods [47, 48], showed distinct differences. Following light exposure, arrestin-1 displayed the expected directional movement from the inner photoreceptor compartments into the outer segments in both wild-type and *Clrn1* KO retinas (Fig 4). Although the direction of arrestin-1 redistribution is similar in both types of mouse retinas, the immunostaining pattern in the *Clrn1* KO mice was different than wild-type, with more arrestin-1 remaining in the outer plexiform layer. We used a semiquantitative evaluation of arrestin-1 immunoreactivity to determine if there were significant changes in arrestin-1 OPL localization in KO relative to wild-type retinas (Fig 4B). The relative signal intensity of arrestin1 in the OPL was significantly greater in the *Clrn1* KO group compared to wild-type control mice (17.17±0.93 in KO vs. 10.45±0.52 in WT, p<0.0001). Light-exposed wild-type mice displayed a weak residual fluorescence staining signal in the OPL, suggesting that arrestin-1 is never entirely removed from its synaptic location following light exposure. However, the majority of *Clrn1* KO mice examined under identical conditions had a much higher arrestin-1 immunoreactive signal at regions comprising the photoreceptor portion of the OPL. Rod transducin-α however translocated similarly towards the inner compartments and OPL of photoreceptors in both *Clrn1* KO and wild-type of mice (S3 Fig). In contrast to arrestin-1, the mean relative intensity of α-transducin immunoreactivity in the OPL of *Clrn1* KO mice was not significantly different from the wild-type (16.95±1.57 in KO vs 15.97±1.63 in WT, p = 0.659, Fig 4B). In conclusion, α-transducin translocation in rods is not affected by the lack of CLRN1 under our experimental light conditions.

**Fig 5:**
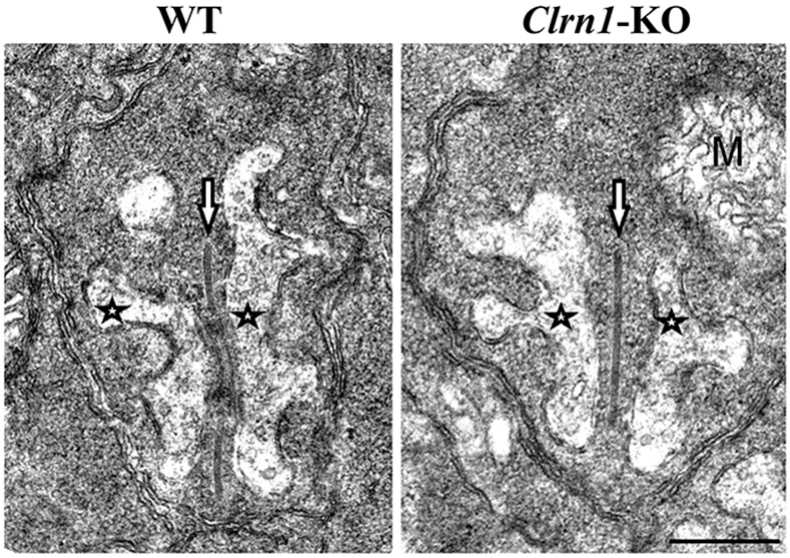
Representative electron microscopic images showing photoreceptor ribbon synapse ultrastructure in WT (left panel) and *Clrn1* KO (right panel) mice. Arrows point to synaptic ribbons and asterisks indicate horizontal cells; M, mitochondria. Scale bar, 500 nm.

The finding that more arrestin-1 remained confined within the OPL of *Clrn1* KO mice relative to wild-type controls following light exposure prompted us to examine in more detail the ultrastructure of the ribbon synapses here using electron microscopy. The presynaptic ribbon of photoreceptor cells constitutes a large, dynamic, electron dense structure that is covered by hundreds of synaptic vesicles and displays a high degree of structural plasticity [55]. No obvious structural differences in the ribbon synapses of wild-type and *Clrn1* KO mice were observed (Fig 5), suggesting that the ultrastructural morphology of photoreceptor synapses is not affected by CLRN1 deficiency.

## Discussion

CLRN1 was originally suggested to have a synaptic role based on its sequence homology with stargazin, also a tetraspanin, which regulates the synaptic targeting of glutamate receptors in the brain [9]. In the cochlea, CLRN1 was shown to be important for maintaining the stability of the F-actin rich stereocilia [31–33], as well as playing a role in the synaptic maturation of hair cells [56]. However, the role of CLRN1 in the retina remains poorly understood mainly because USH3 mouse models have normal retinal function and morphology, and reports on its endogenous cellular localization are conflicting. Previous studies indicate that *Clrn1* is expressed either in the inner or outer retina, depending on the method used for analysis. One study found that *Clrn1* mRNA is expressed in a developmentally regulated manner in Müller cells, and its expression declines to undetectable levels from the inner retina as determined by *in situ* hybridization [33]. Interestingly, we determined that a 2kb region from the endogenous mouse *Clrn1* promoter resulted in non-uniform transgene distribution, concentrated mainly at the injection site in photoreceptors and RPE cells, with undetectable expression levels in the inner retina. Although this promoter region does not contain all the elements required for the endogenous transcriptional control of *CLRN1* expression in various cell types, its *in vivo* characterization suggests that its activity is considerably weaker than CBA, in agreement with previous cell culture experiments [40], and that CLRN1 may indeed be absent or only expressed at low levels in the inner retina. One study using a custom-made antibody indicated that the protein was expressed at the base of the connecting cilium, inner segment and ribbon synapses of photoreceptors [35]. In contrast, RT-PCR experiments from retinal extracts with or without photoreceptor cells concluded that *Clrn1* mRNA originates exclusively from the inner retina [33]. Siegert *et al*. created an online gene expression profile database by using several transgenic mice and showed that *Clrn1* mRNA was found predominantly in starburst amacrine cells [57]. In the zebrafish, the protein was detected both in the inner and outer retina, with strong expression seen at the lateral contacts between photoreceptor inner segments, outer limiting membrane (OLM), and subapical processes from Müller cells, a region containing proteins involved in cell junctions important for photoreceptor stability [58]. Several USH proteins have been localized at the actin-rich OLM [24], including protocadherin 15 [59], with which CLRN1 was previously shown to interact [60].

In this study, we opted for an AAV-based approach to express a CLRN1-Venus fusion protein and examine its distribution by direct imaging of Venus fluorescence in mouse retinal sections. We show that following AAV-mediated delivery, a variety of cells can support CLRN1 expression, including RPE, photoreceptors, ganglion, amacrine, and Müller glia. This is in contrast to the behavior of other AAV-expressed transgenes described previously, in which a similar CBA promoter was used and the vector targeted multiple cell types [61]. Those studies showed that following AAV transfer, transgene expression usually occurred only within those cells from which it was naturally derived, a phenomenon thought to result from the instability of a specific mRNA or protein within the environment of non-endogenous cells [42, 61, 62]. The presence of AAV-expressed CLRN1 in a variety of cell types therefore suggests that off-target cells are capable of producing and maintaining this protein when using a ubiquitous promoter, which has important implications for the design of USH3 gene therapy studies, and for developing a safe *CLRN1* vector delivery system to prevent the photoreceptor cell degeneration in USH3 patients. Taken together, previous studies based on RT-PCR indicate that CLRN1 is endogenously expressed at low levels [33, 57]. In order to avoid potential toxic effects associated with overproduction of CLRN1 in photoreceptor cells, the right combination of AAV vector serotype, titer, and promoter needs to be identified.

Previous expression studies in HEK293 cells demonstrated that CLRN1 is a plasma membrane protein, showing little colocalization with ER markers, and is enriched in specific cell membrane regions including microvilli, lamellipodia, and cellular protrusions rich in F-actin [29, 31]. In our *in vivo* study, we found that the AAV-expressed CLRN1 is present at the inner segments and synaptic regions of photoreceptor cells. Subcellular localization of proteins is essential for their biological function, and, as shown by previous studies, photoreceptor membrane proteins lacking specific targeting information within their amino acid sequence tend to accumulate predominantly in the outer segment compartment [51, 63]. Consequently, CLRN1 may contain distinct amino acid motifs for its specific targeting to inner photoreceptor compartments, avoiding trafficking towards the outer segment.

In the retina, the USH protein network is thought to play a major role in the intracellular trafficking processes within photoreceptor cells through the connecting cilium [25, 26, 64]. In addition, recent studies have shown that the USH interactome is molecularly linked to other proteins whose mutations lead to ciliopathies, including RPGR and CEP290 [65–67]. Many USH proteins have also been localized either pre- or post-synaptically at the specialized ribbon synapses of photoreceptors and second order neurons, suggesting a potential role for these proteins in maintaining the synaptic plasticity and transport of cargo vesicles to the synapse [17, 18, 20, 23, 68]. In spite of their presence at the OPL, no functional evidence of photoreceptor synaptic defects resulting from deficiency in any USH protein was found in the currently available USH animal models [69], including our *Clrn1* KO mice shown here [33]. The scaffold proteins SANS and whirlin have been shown to assemble a protein network composed of USH1 and USH2 members at the periciliary region, situated at adjacent membranes of the inner segment collar and the connecting cilium, with SANS linking the macromolecular complex to the microtubule cytoskeleton [26]. One study showed that CLRN1 can interact directly with the USH1F protein, protocadherin-15 (USH1F protein) [60]. CLRN1 may thus play a structural role in the ciliary–periciliary region of photoreceptors as a member of the USH interactome, potentially impacting protein transport processes as well. The increase of arrestin-1 remaining in the OPL upon light exposure in *Clrn1* KO compared to wild-type controls is consistent with a delay in the arrestin-1 translocation towards the outer segments. In the dark, arrestin-1 is anchored within the inner compartments of photoreceptors through interactions with other proteins [70]. These interactions include microtubules, which are abundant not only within the synaptic region, but also within the inner segments of photoreceptor cells [71, 72]. Cytoskeletal elements, such as microfilaments and microtubules, play both structural and transport roles in photoreceptor cells, in addition to maintaining the morphology and functional plasticity of ribbon synapses [73]. CLRN1 was shown to exhibit a potential association with either tubulin or F-actin in previous studies [29, 35] and thus, CLRN1 deficiency may destabilize the network of proteins that structurally support a normal cytoskeleton in photoreceptor cells.

In summary, we show that a variety of cells can support CLRN1 expression in the retina following AAV delivery, and this has important implications for designing gene therapy studies. We found that the compartmentalization of AAV-expressed CLRN1 in photoreceptors is similar to that of known Usher proteins, localizing primarily to the inner segment region and outer plexiform layer. In view of its apparently low levels of endogenous expression as determined by previous studies, vector optimization remains a critical aspect for developing safe therapies for USH3 disorder.

## Supporting Information

S1 Fig Expression of mCherry as driven by a 2kb mouse *Clrn1* promoter following subretinal vector delivery. Left panel: Retinal cross-section showing that both photoreceptors and RPE cells are transduced (Scale bar, 20 μm). Right panel: low magnification image showing an overview of the entire retina. Note that expression is mainly localized at the injection site. The image was purposefully overexposed to detect expression.
(TIF)

S2 Fig Immunofluorescent localization of arrestin-1 and α-transducin in *Clrn1* KO and WT retinal sections in the dark. Compartmentalization of arrestin-1 (red) and α-transducin (green) in dark-adapted conditions are similar in *Clrn1* KO and WT retinal sections. Arrestin-1 is localized to rod inner segments, ONL, and OPL (left panels), while α-transducin is found in the outer segments of both WT and *Clrn1* KO mice (middle panels).
(TIF)

S3 Fig Distribution of α-transducin (green) following light exposure. Images show the α-transducin movement from the OS towards the inner parts of photoreceptor cells, rod inner segments (IS), ONL, and OPL, in both WT and *Clrn1* KO mice, and the corresponding fluorescence signal intensity profiles through the photoreceptor layer. Nuclei are stained blue with DAPI.
(TIF)
