# Supplementary figures and images for: AAV-Mediated Clarin-1 Expression in the Mouse Retina: Implications for USH3A Gene Therapy
Source: PLoS One. 2016 Feb 16;11(2):e0148874. doi: 10.1371/journal.pone.0148874 (PMC4755610; doi:10.1371/journal.pone.0148874)

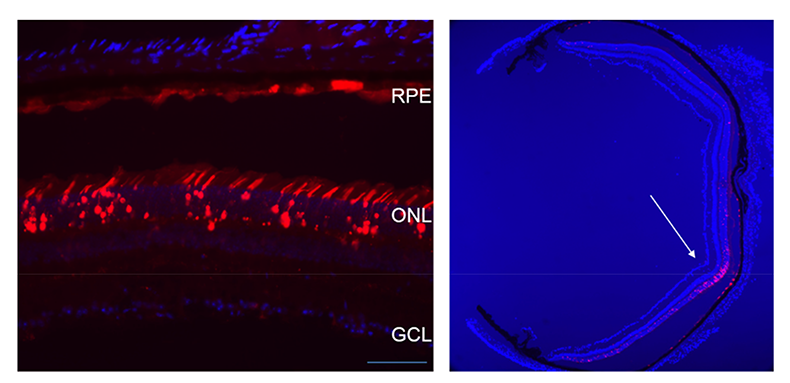

Supplement: S1 Fig — Left panel: Retinal cross-section showing that both photoreceptors and RPE cells are transduced (Scale bar, 20 μm). Right panel: low magnification image showing an overview of the entire retina. Note that expression is mainly localized at the injection site. The image was purposefully overexposed to detect expression. (TIF) [file pone.0148874.s001.tif]

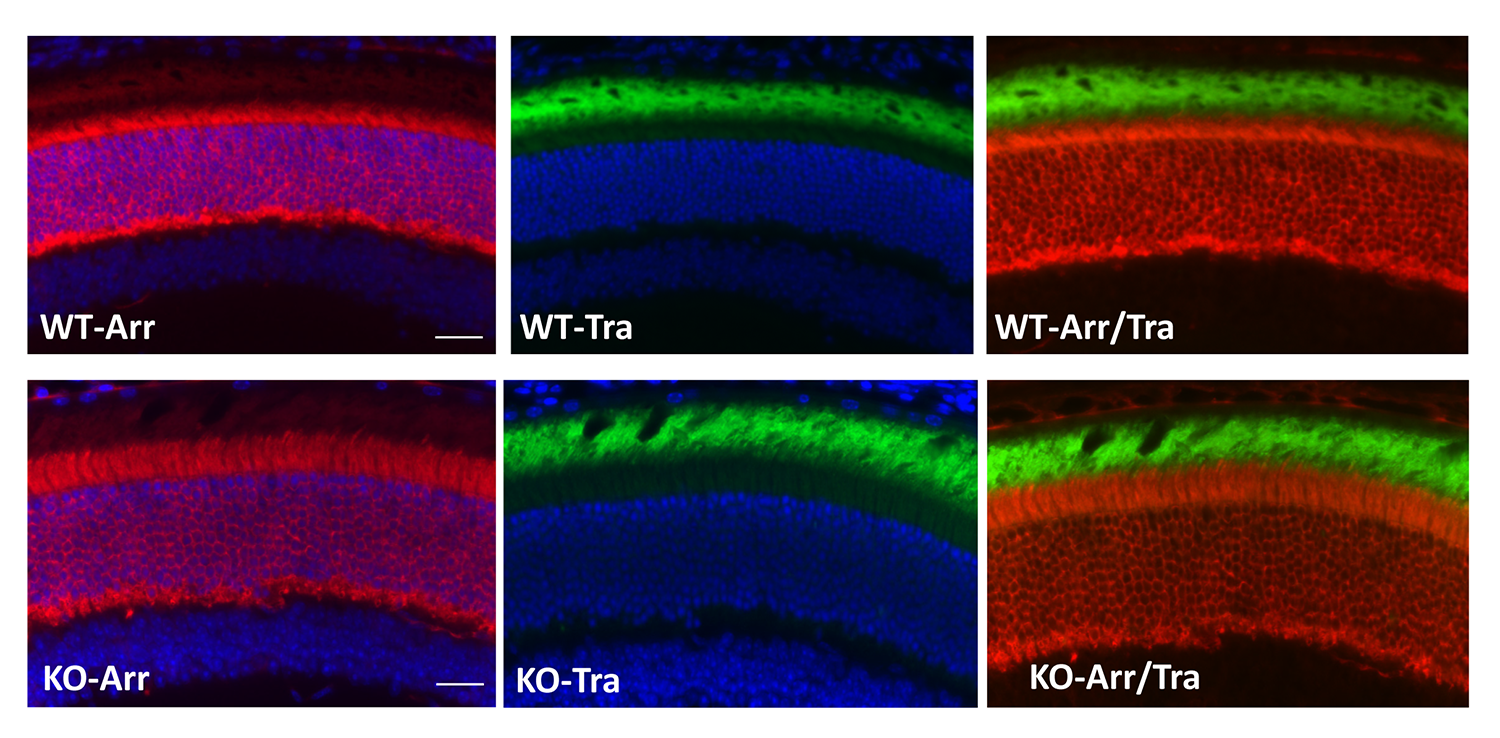

Supplement: S2 Fig — Compartmentalization of arrestin-1 (red) and α-transducin (green) in dark-adapted conditions are similar in Clrn1 KO and WT retinal sections. Arrestin-1 is localized to rod inner segments, ONL, and OPL (left panels), while α-transducin is found in the outer segments of both WT and Clrn1 KO mice (middle panels). (TIF) [file pone.0148874.s002.tif]

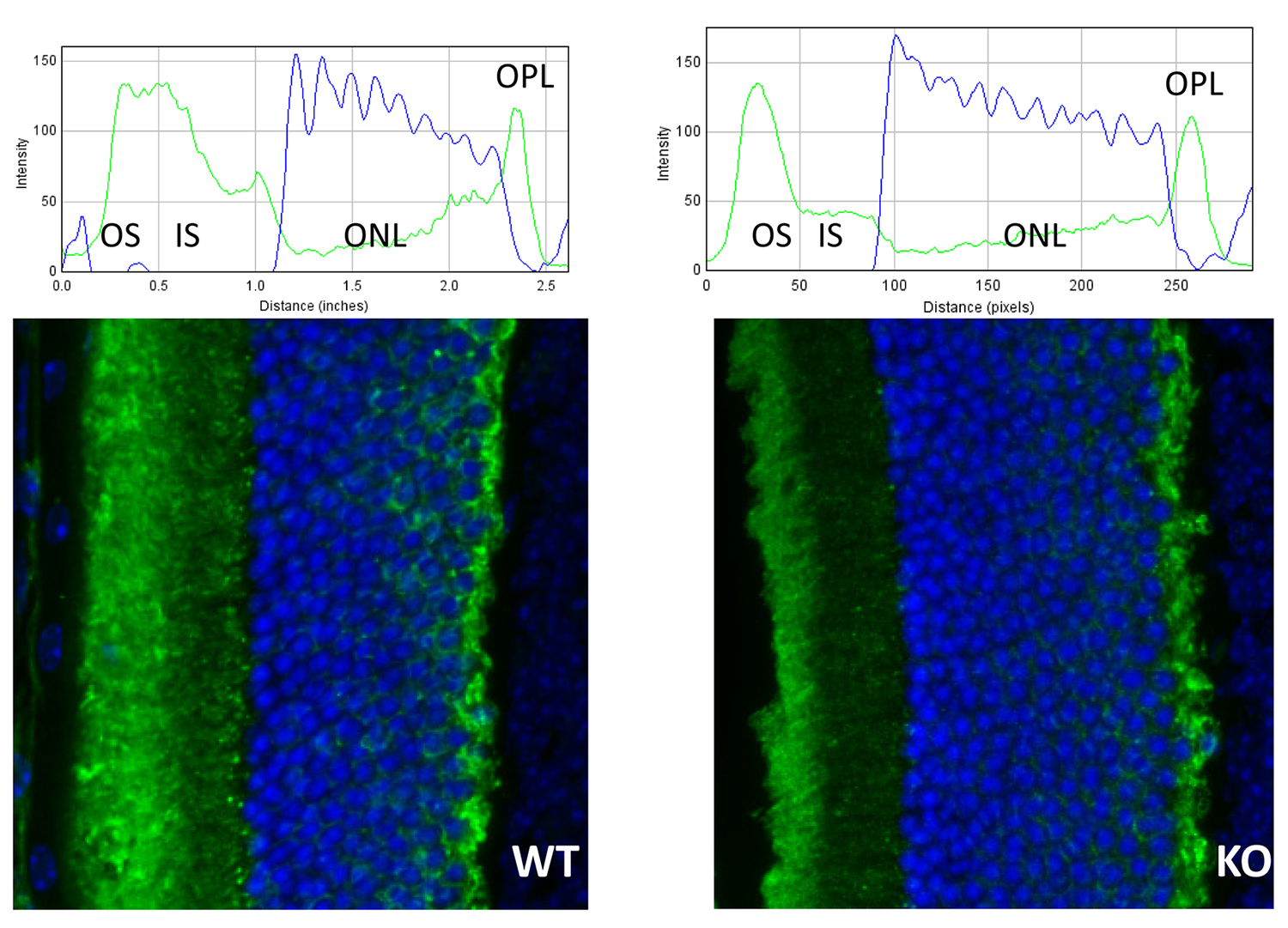

Supplement: S3 Fig — Images show the α-transducin movement from the OS towards the inner parts of photoreceptor cells, rod inner segments (IS), ONL, and OPL, in both WT and Clrn1 KO mice, and the corresponding fluorescence signal intensity profiles through the photoreceptor layer. Nuclei are stained blue with DAPI. (TIF) [file pone.0148874.s003.tif]
